# ER network dynamics are differentially controlled by myosins XI-K, XI-C, XI-E, XI-I, XI-1, and XI-2

**DOI:** 10.3389/fpls.2014.00218

**Published:** 2014-05-21

**Authors:** Lawrence R. Griffing, Hongbo T. Gao, Imogen Sparkes

**Affiliations:** ^1^Biology Department, Texas A&M UniversityCollege Station, TX, USA; ^2^Biosciences, College of Life and Environmental Sciences, Exeter UniversityExeter, UK

**Keywords:** endoplasmic reticulum, movement, myosin, persistency mapping, dynamics, remodeling

## Abstract

The endoplasmic reticulum (ER) of higher plants is a complex network of tubules and cisternae. Some of the tubules and cisternae are relatively persistent, while others are dynamically moving and remodeling through growth and shrinkage, cycles of tubule elongation and retraction, and cisternal expansion and diminution. Previous work showed that transient expression in tobacco leaves of the motor-less, truncated tail of myosin XI-K increases the relative area of both persistent cisternae and tubules in the ER. Likewise, transient expression of XI-K tail diminishes the movement of organelles such as Golgi and peroxisomes. To examine whether other class XI myosins are involved in the remodeling and movement of the ER, other myosin XIs implicated in organelle movement, XI-1 (MYA1),XI-2 (MYA2), XI-C, XI-E, XI-I, and one not, XI-A, were expressed as motor-less tail constructs and their effect on ER persistent structures determined. Here, we indicate a differential effect on ER dynamics whereby certain class XI myosins may have more influence over controlling cisternalization rather than tubulation.

## Introduction

The endoplasmic reticulum (ER) is a polygonal network that rapidly interconverts between tubular and cisternal forms, with tubules also growing, shrinking, and undergoing lateral sliding to form junctions, and cisternae undergoing expansion and shrinkage (Griffing, 2010; Sparkes et al., 2011). Whilst ER network dynamics are mainly controlled by actin and myosin (Quader et al., 1987; Liebe and Menzel, 1995; Sparkes et al., 2009a; Ueda et al., 2010), ER morphology and structure are also governed by additional factors such as reticulons and RHD3 implicated in ER membrane curvature and homotypic fusion (Sparkes et al., 2011; Zhang and Hu, 2013). Quantifying network dynamics is challenging because there are at least two levels at which movement takes place; (1) flow of protein within the lumen and on the surface of ER tubules and cisternae, as well as (2) remodeling through translocation and transformation of the tubular and cisternal elements themselves. Note, membrane flow can occur even when there is no remodeling (Sparkes et al., 2009a). The former movement, flow within and on the membrane is measured with fluorescent recovery after photobleaching (FRAP) and photoactivation (Sparkes et al., 2009a) as well as with optical flow image processing (Ueda et al., 2010; Stefano et al., 2014). But it is the latter form of movement, ER remodeling, which is analyzed here, starting from the perspective that there are relatively persistent features of the ER upon which change agents act. Hence, an approach to visualize and measure the relatively static or persistent regions in the network has been developed, persistency mapping (Sparkes et al., 2009a). Persistency mapping is an image processing technique that includes morphometric processing to separate tubules from cisternae and visualizes the relative movement and change of these ER elements as the ER remodels. The less-moving, more persistent fraction of the ER tubules and cisternae is then quantified. It is tempting to assign functions of some of the persistent structures of the ER, such as the potential anchor sites (Sparkes et al., 2009a,b) which may be regions where traffic between the plasma membrane and the ER can take place or/and could act as anchors to tether the ER required for spread throughout the cortex. A role in tethering or anchoring the ER to the plasma membrane was alluded to by optical tweezer experiments whereby a trapped Golgi body was used to stably remodel the ER around small islands of ER (Sparkes et al., 2009b). These experiments also highlighted that there is a physical connection between Golgi bodies and the ER bringing into question whether Golgi and ER dynamics are coordinated or are independent of one another? The common network upon which these interactions play out is the actin cytoskeleton.

Organelle movement in interphase plant cells is largely driven by actin and myosin, however the specificity of myosin-organelle interactions is poorly understood (Madison and Nebenführ, 2013 and references therein). In plants, organelle positioning and movement are correlated with responses to extracellular stresses such as pathogen ingress, wounding, and cadmium toxicity (Takemoto et al., 2003; Lipka et al., 2005; Hardham et al., 2008; Rodriguez-Serrano et al., 2009). At present, the precise role that organelle dynamics play in such processes is unclear. The best characterized study of the functional role of organelle movement is that of chloroplast repositioning upon high light, a response required to prevent photodamage (Wada et al., 2003). In addition there is a correlation between the morphology of the ER and the level of secretion with cisternal ER being more prevalent in cells producing more protein (Stephenson and Hawes, 1986; Ridge et al., 1999). Treating cells with latrunculin B to depolymerize actin results in a cessation of movement of spheroid organelles such as Golgi (Boevink et al., 1998; Nebenführ et al., 1999), mitochondria (Van Gestel et al., 2002; Zheng et al., 2009), and peroxisomes (Jedd and Chua, 2002; Mano et al., 2002; Mathur et al., 2002), and produces a relatively static ER network (Quader et al., 1987; Liebe and Menzel, 1995; Sparkes et al., 2009a) with larger cisternae, indicating that an actively remodeling system affects the global morphology of the ER. The movement of Golgi, peroxisomes and mitochondria appear to be controlled by a subset of class XI myosins; XI-C, XI-E, XI-I, XI-K, XI-1, and XI-2 (Avisar et al., 2008, 2009, 2012; Peremyslov et al., 2008, 2010; Prokhnevsky et al., 2008; Sparkes et al., 2008, 2009a). Dominant negative tail domain expression of these different myosins shows some quantitative differences in the reduction of movement produced by each member of this subset, but the results do not preclude the simple interpretation that one myosin form may control the movement of several different organelles. Double, triple and quadruple *Arabidopsis* mutants in myosins XI-K, XI-1, XI-2, and XI-I have reduced organelle dynamics and display gross morphological defects (Prokhnevsky et al., 2008; Peremyslov et al., 2010; Ojangu et al., 2012). Although myosin XI-K has been shown to change ER form and dynamics (Sparkes et al., 2009a; Ueda et al., 2010), work with mutants of XI-1, and XI-2, shows that they have little effect on their own, but enhance the effect of XI-K when double or triple mutants are analyzed. Here, we further explore whether these different subclasses of myosin XI that reduce spheroid organelle mobility differentially affect the movement and remodeling of the ER network. Remodeling is assessed by quantifying the static elements in the network which should increase if motor activity is required to drive changes.

## Materials and methods

### Plant material and constructs

*Nicotiana tabacum* plants were grown according to Sparkes et al. (2005). Fluorescent protein fusion constructs including the ER marker GFP-HDEL (Batoko et al., 2000), and mRFP-myosin XI-K, XI-I, XI-1, XI-2, XI-E, XI-C, XI-A tail domains (Sparkes et al., 2008; Avisar et al., 2009) were all infiltrated according to Sparkes et al. (2006) with an optical density of 0.1 except for GFP-HDEL, which required 0.04 optical density. Expression was analyzed 3 days following inoculation.

### Protein extraction and western blotting

Total proteins were extracted according to Gao et al. (2013). 0.5 g of tobacco leaf material 3 days post infiltration were ground in PEB (50 mM Tris–HCl, pH 7.5, 150 mM NaCl, 1 mM EDTA, 1% TritonX-100 plus protease inhibitors) and then centrifuged. Equal volumes of extract were separated by 10% SDS-PAGE and blotted onto PVDF membrane (Pall). mRFP fusions were detected using anti-mRFP primary antibody (Abcam) and HRP-conjugated goat anti-rabbit secondary antibody (Abcam). Chemiluminescence reaction was performed using ECL substrate (Pierce) followed by film exposure.

### Sample preparation and image acquisition

The ER in the outermost cortical region of adaxial leaf epidermal pavement cells was imaged. Dual imaging of mRFP and GFP was done using multi-tracking in line switching mode on a Zeiss LSM510 Meta confocal microscope. GFP was excited with a 488 nm argon laser and mRFP with a 543 nm laser and their emissions detected using a 488/543 dichroic mirror and 505–530 and 560–615 nm band pass filters, respectively. All imaging was carried out using a 63 × 1.4 numerical aperture oil immersion objective.

### Persistency mapping the cortical ER in tobacco epidermal cells

For persistency mapping, time-lapse images of GFP-HDEL were captured using a 2–3 μm pinhole, 512 × 512 pixel resolution and 2.3× digital zoom. To reduce noise, 4× line averaging was used. The scan rate was increased by imaging a 955–960 μm^2^ region of interest (ROI), so that 50 frames per 80 s were captured (0.63 frames/s). For samples where cells were coexpressing a fluorescent myosin tail domain and GFP-HDEL, coexpression was verified before time lapse imaging of the GFP-HDEL alone was performed.

Persistency maps were generated in Image J (version 1.45s, Wayne Rasband, National Institute of Health, Bethesda, MD) as described in Sparkes et al. (2009a) with the following modifications. As shown in the corrected tubule persistency maps in Figures 2, 3, the persistent tubule subset was corrected by subtracting regions containing cisternae prior to making persistent tubule counts. This was done by directly subtracting the morphologically opened binary sum images from the morphologically closed skeletonized binary sum images. In the resulting image sets, only those with a projected area > 0.2 μm^2^ were counted (excludes tubules less than 1 μm long, assuming a 200 nm projection of an individual tubule). This disconnected some of the tubules because punctae or small cisternae often occur at tubule junctions, producing shorter tubules of smaller percentage area than previously described. As shown in the corrected cisternal persistency maps of Figures 5, 6, the persistent cisternal subset was corrected by subtracting regions that contained continuous flow (i.e., subtracting the sum of the 5-frame differences from the cisternal persistency map). Quantitation of the most persistent tubules, and cisternae was done, normalizing each movie to the total membrane area imaged in the percentage area and number/100 μm^2^ values. Analysis of mesh size and mesh number per cytoplasmic volume was done with manual selection of the region containing in-focus signal for each movie, then measuring the number and area of selected mesh regions for each frame of every movie. The cytoplasmic volume was estimated as the area of the region containing in-focus signal times the z-dimension optical section acquired by the confocal microscope. Analysis of the size classes of persistent cisternae was done by making a binned histogram of the different sizes of persistent cisternae for the entire collection of movies acquired for each treatment. Note, highly persistent regions are defined as being present (persisting) for more than 23 s within the movie. Statistical comparison of the data was done using the 64 bit version of R, 3.0.2 (http://www.r-project.org/) using ANOVA and the Tukey HSD two-way comparison of means at 95% confidence interval. Each analysis is based on data generated from 15 movies in total taken from separate cells from three independent experiments.

Displaced frame difference images were made by subtracting every fifth frame, summing the differences between all frames, and dividing that value by an estimated cytoplasmic volume (area containing signal in a 3 μm optical slice) for each movie.

## Results

### Myosin tail domains affect ER morphology

**Figure 1:**
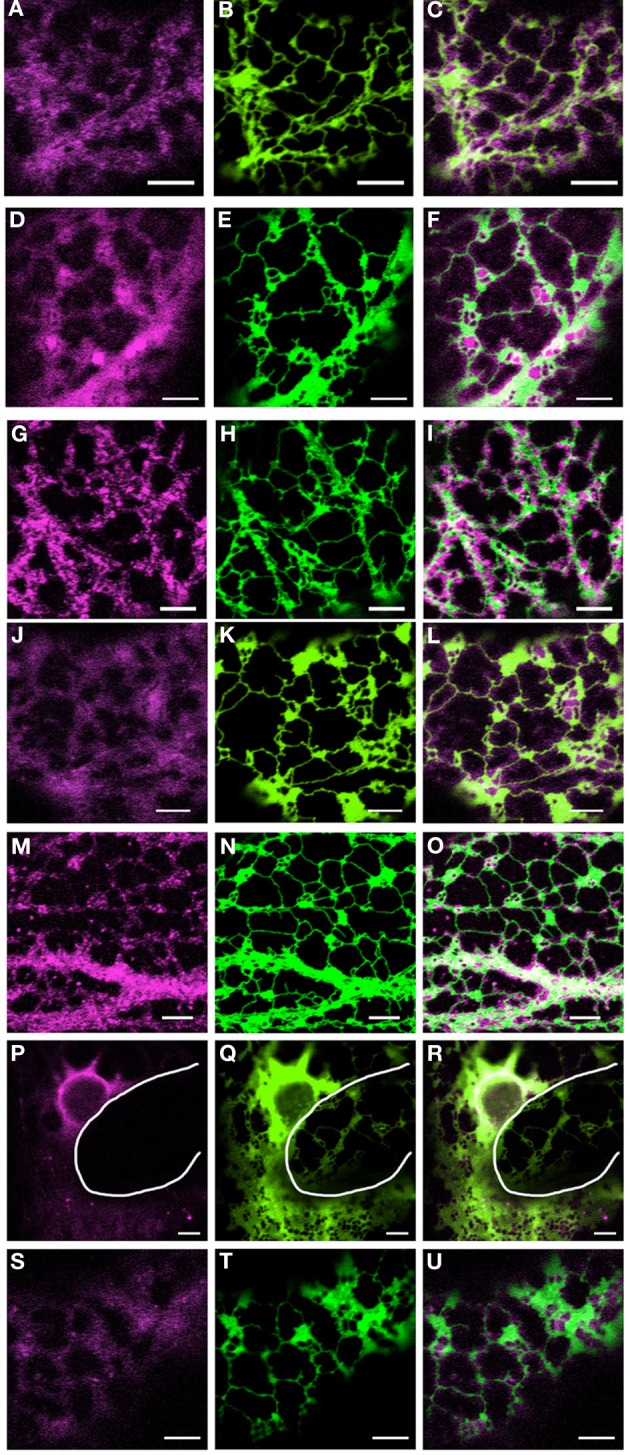
**Coexpression of myosin tail domains with an ER marker**. Representative images of cells coexpressing GFP-HDEL (green) and mRFP myosin tail domain fusions [magenta; XI-A, **(A–C)**; XI-1, **(D–F)**; XI-2, **(G–I)**; XI-C, **(J–L)**; XI-E, **(M–O)**; XI-I, **(P–R)**; XI-K, **(S–U)**]. White line in panels **(P–R)** highlights the cell boundary between neighboring cells. Scale bar 5 μm.

As previously reported, transient expression of mRFP-myosin tail fusions in tobacco leaf epidermal cells are present in puncta or diffusely throughout the cytoplasm, with XI-I collating to the nucleus and motile puncta (Figure 1; Avisar et al., 2009). A similar perinuclear localization for both full length and tail fusions of XI-I in Arabidopsis and tobacco respectively were reported by Tamura et al. (2013). Here, we show how these fusions relate to, and affect the morphology and remodeling of the ER. Figure 1 highlights that whilst these fusions do not solely collocate to the surface of the ER, they appear to differentially affect the level of ER cisternalization with XI-I tail resulting in the largest observable regions of cisternal ER (Figures 1P–R). This phenomenon is clearly visible when comparing the morphology of the ER in two adjacent cells where one has no detectable levels of mRFP-XI-I tail fusion (Figures 1P–R, white line defines cell boundary). These still images do not convey whether myosin tail expression affects the dynamic remodeling of the ER and so time lapse movies were taken and dynamics quantified.

### Myosin tail domains affect global ER remodeling

Movies were taken using consistent settings allowing comparisons between the effects of the myosin tail domains on ER dynamics to be drawn. In addition, the same settings were used to detect mRFP myosin tail fusions to determine whether any effects on ER dynamics were dependent on myosin tail expression level. Western blotting confirmed that the majority of the myosin tail fusions were stable under transient expression in tobacco leaf epidermal cells (Figure S1). Results indicated that whilst XI-A (Movie S2) tail domain didn't appear to affect global ER remodeling compared with control cells only expressing the ER marker (GFP-HDEL, Movie S1), the other tail domains perturbed remodeling to differing extents in a concentration independent manner; XI-2 slowed down movement (Movie S3), XI-I increased the level of cisternal ER which was still relatively mobile (Movie S4), whereas XI-C, XI-E, XI-K, and XI-1 all reduced active remodeling (Movies S5–S9).

### Quantification of the effects of myosin tail domains on ER dynamics

**Figure 2:**
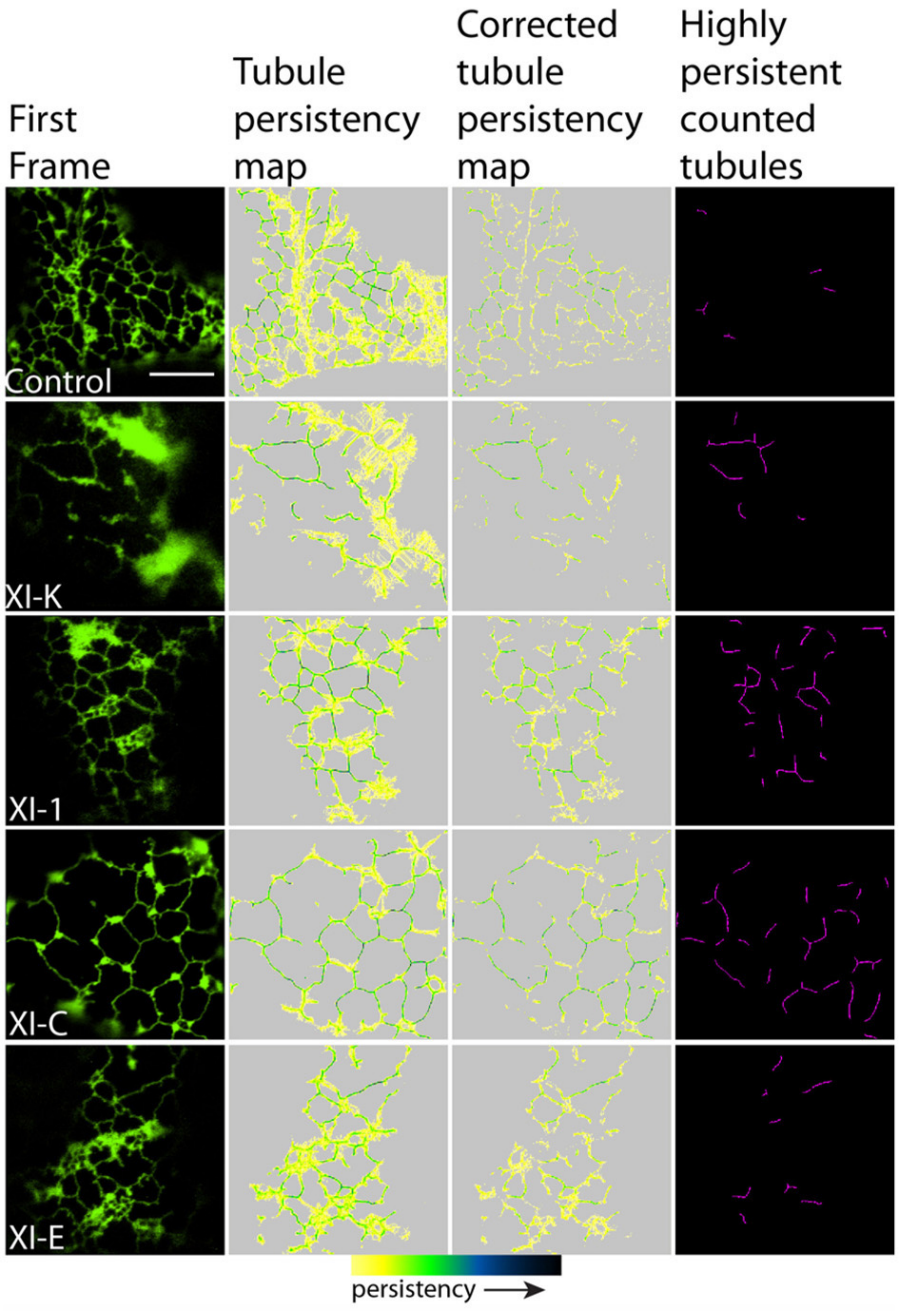
**Effects of XI-K, XI-C, XI-E, and XI-1 myosin tail domains on persistent ER tubules identified with co-expressed GFP-HDEL**. Left-hand panel is the first frame of a 50 frame movie. Middle two panels are tubule persistency expressed as the sum of skeletonized tubules that persist for five frames (7.9 s). The corrected tubule persistency subtracts out contributing skeletons from cisternal regions. The yellow-green-blue-black persistency scale represents persistency from 7.9 to 80 s. Highly persistent tubules are those that are counted for Figure 4, being both greater than 0.2 μm^2^ and persisting for at least a third of total movie time (black to green on the persistency scale with black representing persistency through the whole 80 s and green representing persistency for approximately 23 s). Note, control refers to cells only expressing the ER marker GFPHDEL (green, left hand panel).

**Figure 3:**
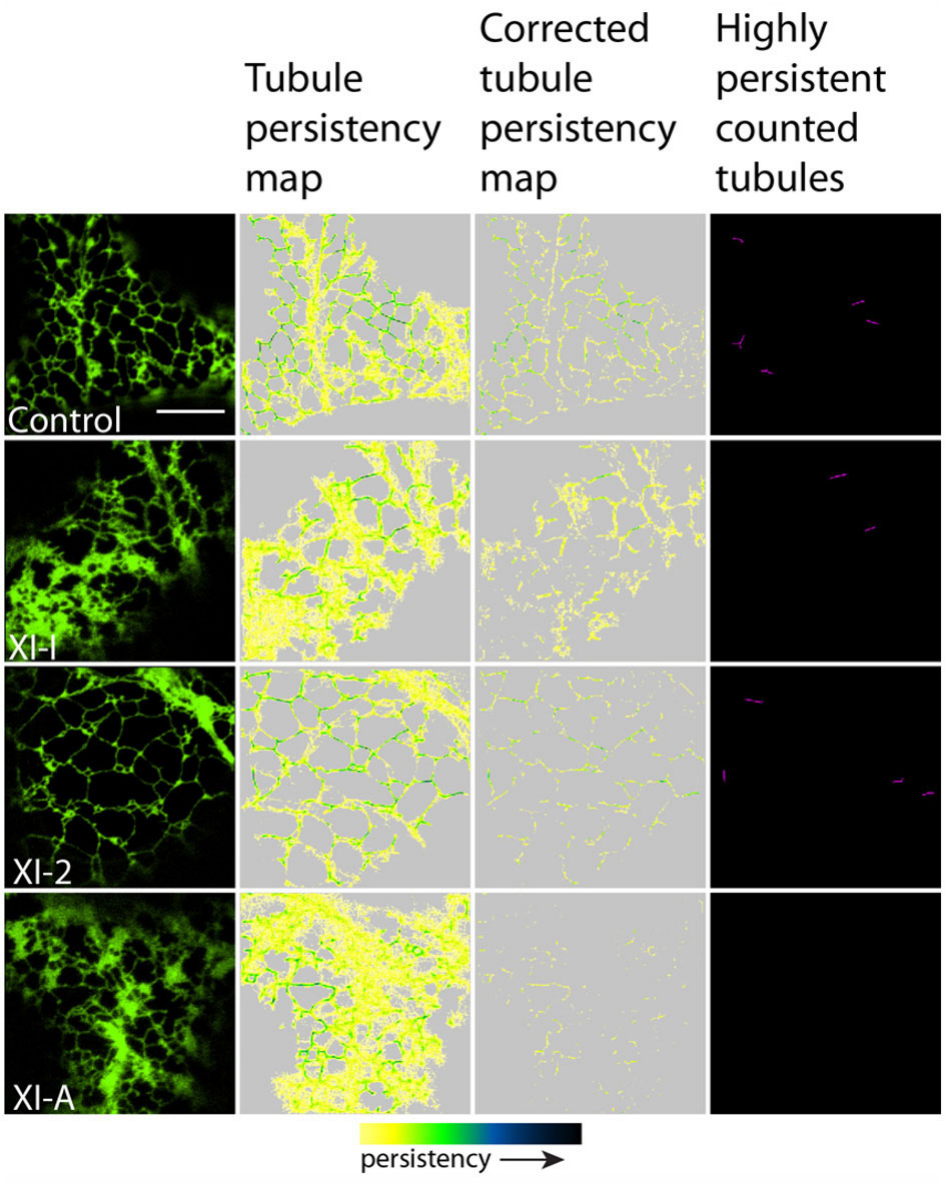
**Effects of XI-2, XI-A, and XI-I myosin tail domains on persistent ER tubules identified with co-expressed GFP-HDEL**. Left-hand panel is the first frame of a 50 frame movie. Middle two panels are tubule persistency expressed as the sum of skeletonized tubules that persist for at least five frames (7.9 s). The corrected tubule persistency subtracts out contributing skeletons from cisternal regions. The yellow-green-blue-black persistency scale represents persistency from 7.9 to 80 s. Highly persistent tubules are those that are counted for Figure 4, being both greater than 0.2 μm^2^ and persisting for at least a third of total movie time (black to green on the persistency scale with black representing persistency through the whole 80 s and green representing persistency for approximately 23 s). Note, control refers to cells only expressing the ER marker GFPHDEL (green, left hand panel).

To quantify the effects of myosin tail domains on ER dynamics we implemented persistency mapping to tease out regions which had become more or less persistent compared with control cells. Persistency mapping can be used to measure the more static elements within the ER network over the given time frame. Morphological elements of the ER under study include tubules, cisternae (areas larger than 0.3 μm^2^) and punctae (areas smaller than 0.3 μm^2^). If the different myosins act as change agents, then their dominant-negative selective inhibition should decrease change, thereby increasing the number or size of the more persistent morphological elements. Therefore, we have chosen here and in prior work (Sparkes et al., 2009a) to measure the more persistent features (green to black in Figures 2, 3, 5, 6) of the persistency map rather than less persistent features (yellow in Figures 2, 3, 5, 6). The analysis here is further refined over prior work by excluding cisternal regions from tubular persistency analysis and by excluding large moving cisternal fields occurring throughout the analysis period (80 s) from the cisternal persistency analysis.

**Figure 4:**
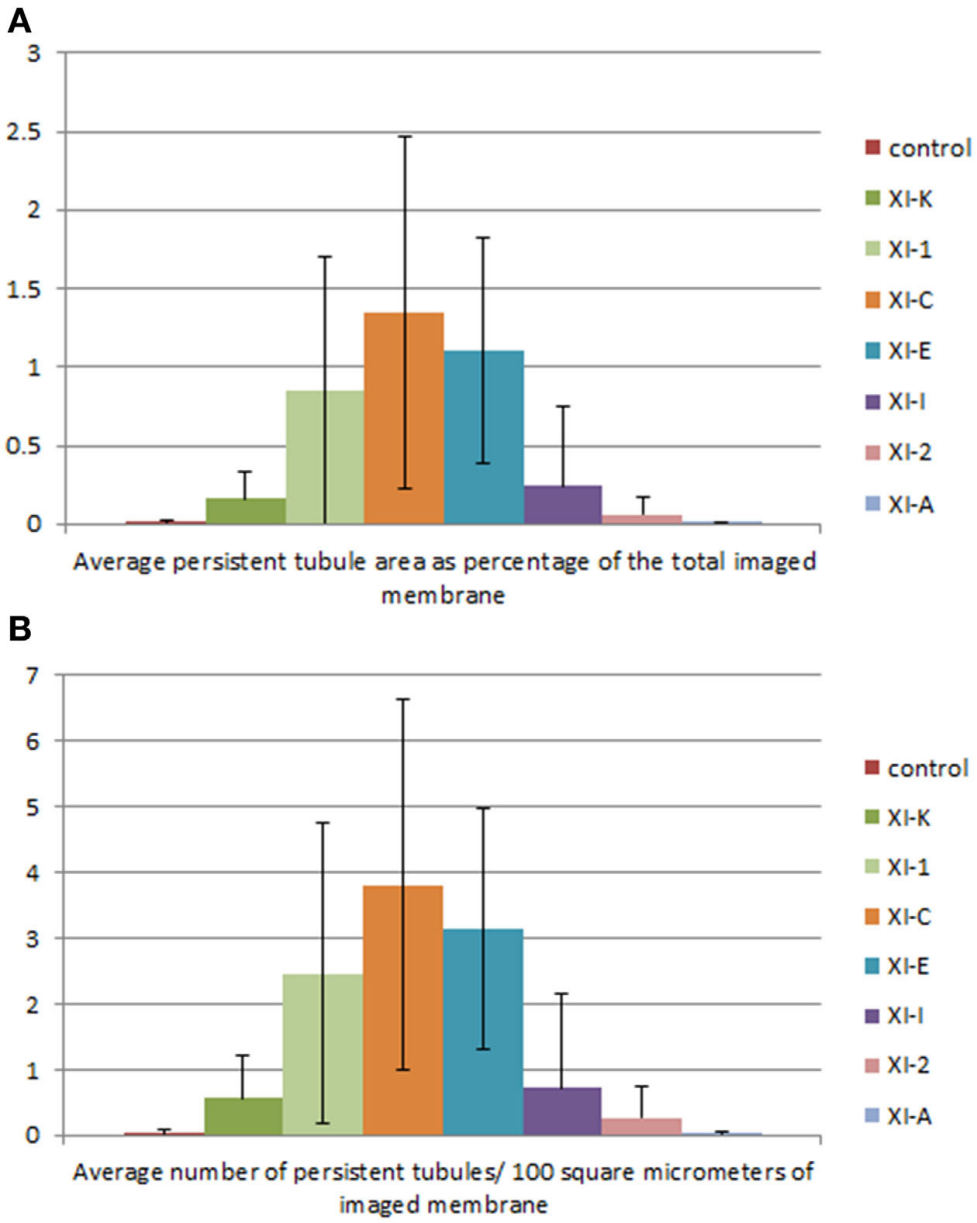
**Quantification of the effects of the myosin tail domains on persistent ER tubules. (A)** Counts of the cumulative percentage area of highly persistent tubules (see Figures 2, 3 for examples). **(B)** Counts of the number of persistent tubules per hundred square micrometers of ER membrane analyzed for all of the myosin subclasses. Error bars represent standard deviation. The level of significance of the difference between the samples is described in Table 1.

**Figure 5:**
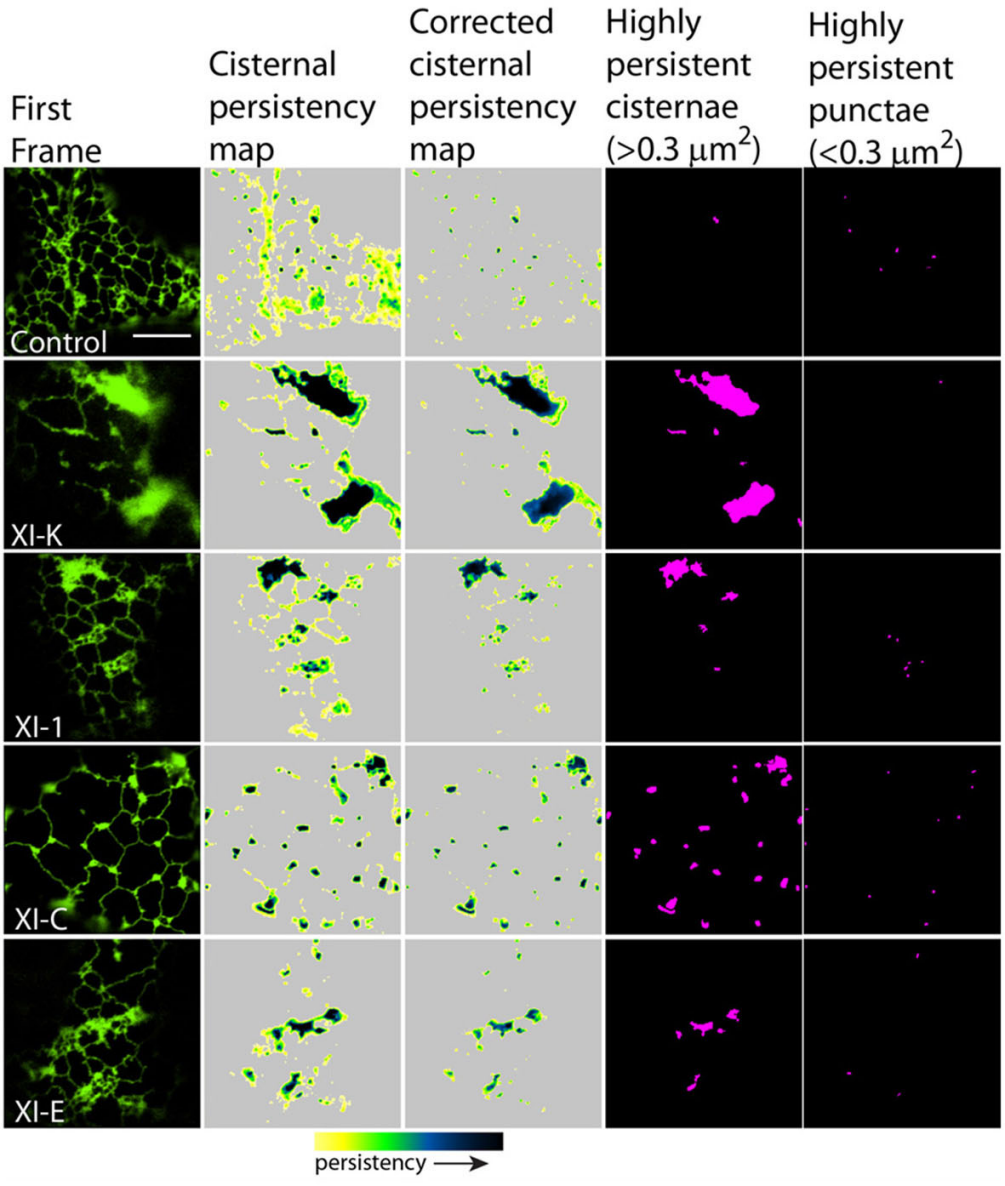
**Effects of XI-K, XI-C, XI-E, and XI-1 myosin tail domains on persistent ER cisternae and punctae identified with co-expressed GFP-HDEL**. Left-hand panel is the first frame of a 50 frame movie. Middle two panels are cisternal persistency expressed as the sum of cisternae (morphometrically separated from tubules with a binary opening operation) that persist for at least five frames (7.9 s). The corrected cisternal persistency subtracts out regions of continuous flow (sum of five frame differences). The yellow-green-blue-black persistency scale represents persistency from 7.9 to 80 s. Highly persistent regions are those that are counted for Figure 7, cisternae being greater than 0.3 μm^2^ and punctae less than 0.3 μm^2^ and both persisting for at least a third of total movie time (black to green on the persistency scale with black representing persistency through the whole 80 s and green representing persistency for approximately 23 s). Note, control refers to cells only expressing the ER marker GFPHDEL (green, left hand panel).

**Figure 6:**
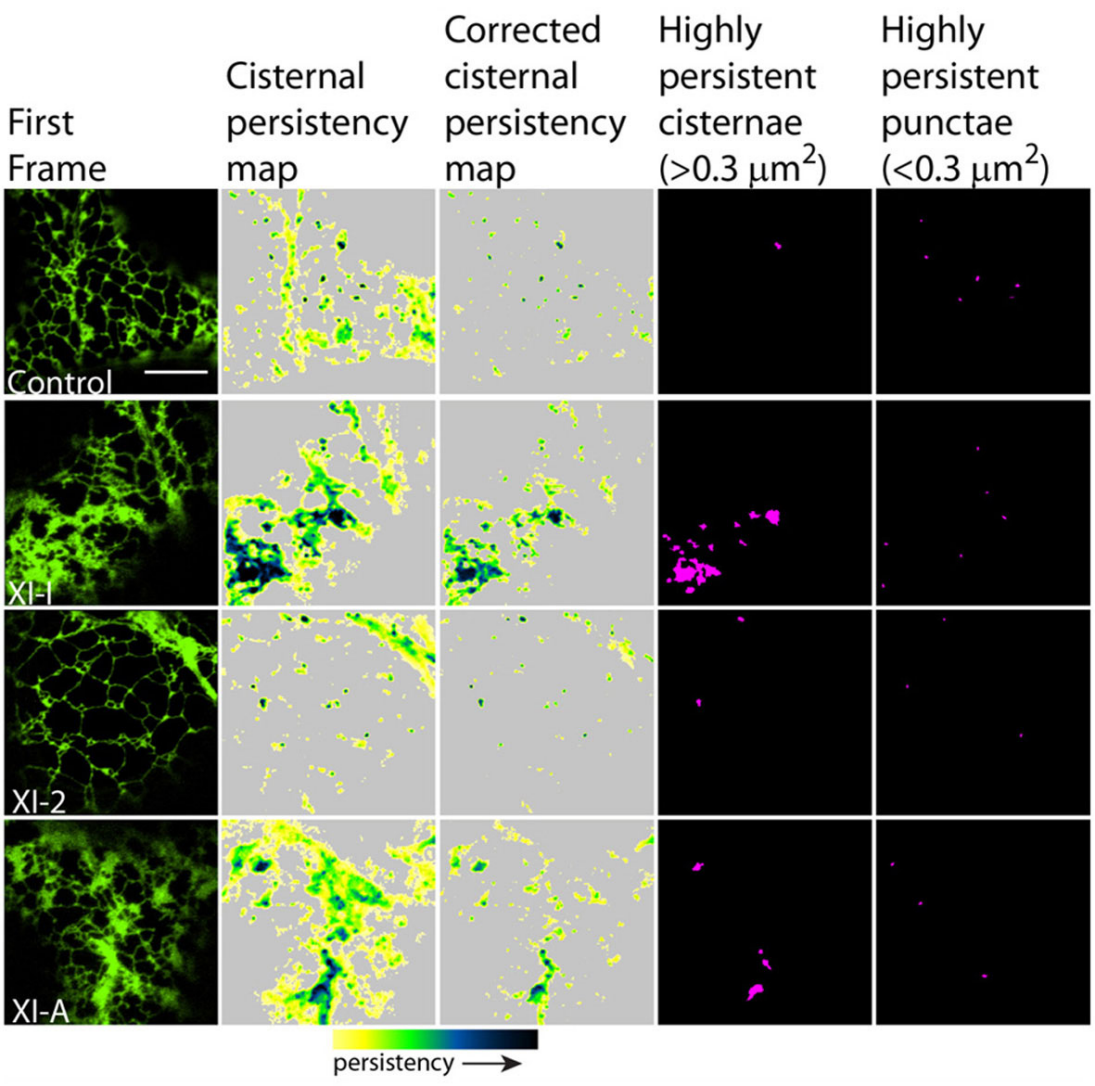
**Effects of XI-2, XI-A, and XI-I myosin tail domains on persistent ER cisternae and punctae identified with co-expressed GFP-HDEL**. Left-hand panel is the first frame of a 50 frame movie. Middle two panels are cisternal persistency expressed as the sum of cisternae (morphometrically separated from tubules with a binary opening operation) that persist for at least five frames (7.9 s). The corrected cisternal persistency subtracts out regions of continuous flow (sum of five frame differences). The yellow-green-blue-black persistency scale represents persistency from 7.9 to 80 s. Highly persistent regions are those that are counted for Figure 7, cisternae being greater than 0.3 μm^2^ and punctae less than 0.3 μm^2^ and both persisting for at least a third of total movie time (black to green on the persistency scale with black representing persistency through the whole 80 s and green representing persistency for approximately 23 s). Note, control refers to cells only expressing the ER marker GFPHDEL (green, left hand panel).

**Figure 7:**
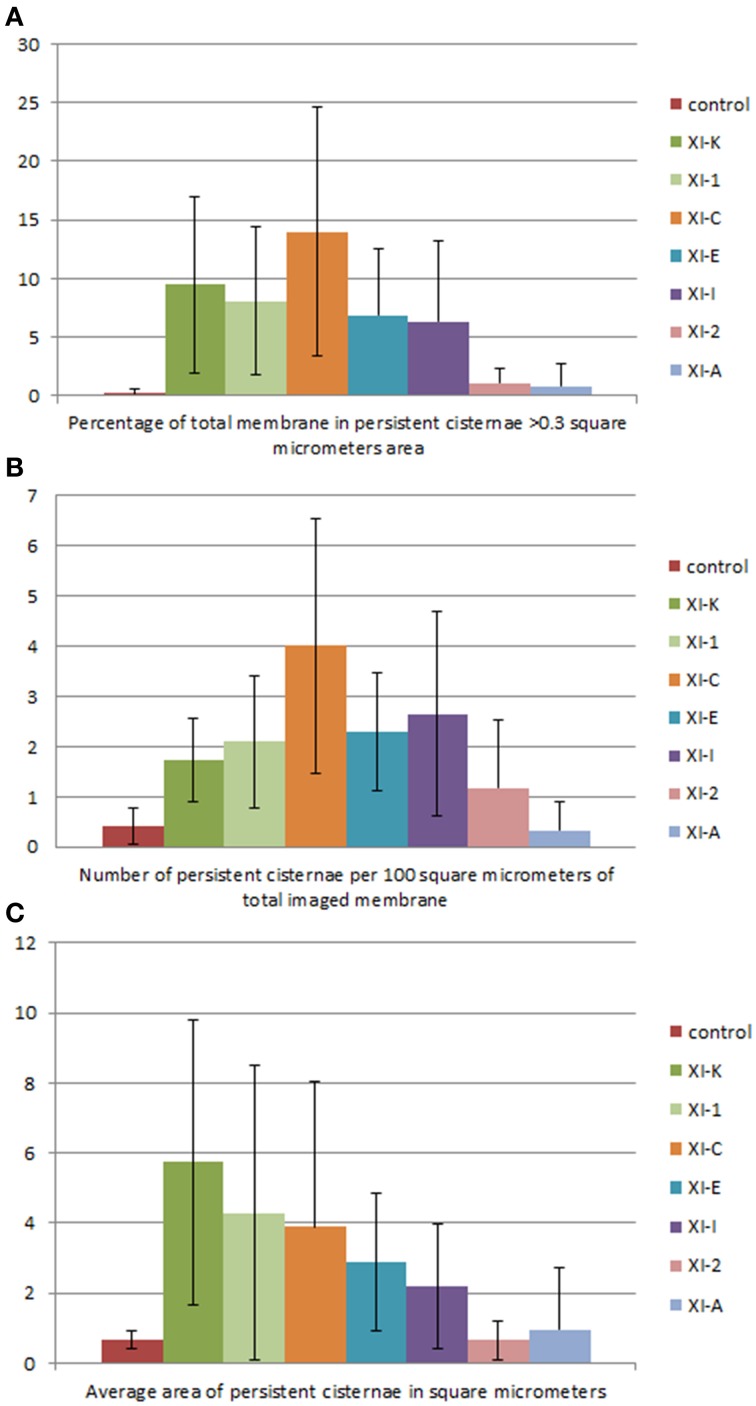
**Quantification of the effects of myosin tail domains on persistent ER cisternae. (A)** Counts of the cumulative percentage area of highly persistent cisternae (see Figures 5, 6 for examples). **(B)** Counts of the number of persistent cisternae per hundred square micrometers of ER membrane analyzed for all of the myosin subclasses. **(C)** Average area of persistent cisternae in square micrometers. Error bars represent standard deviation. The level of significance of the difference between the samples is described in Table 1.

**Figure 8:**
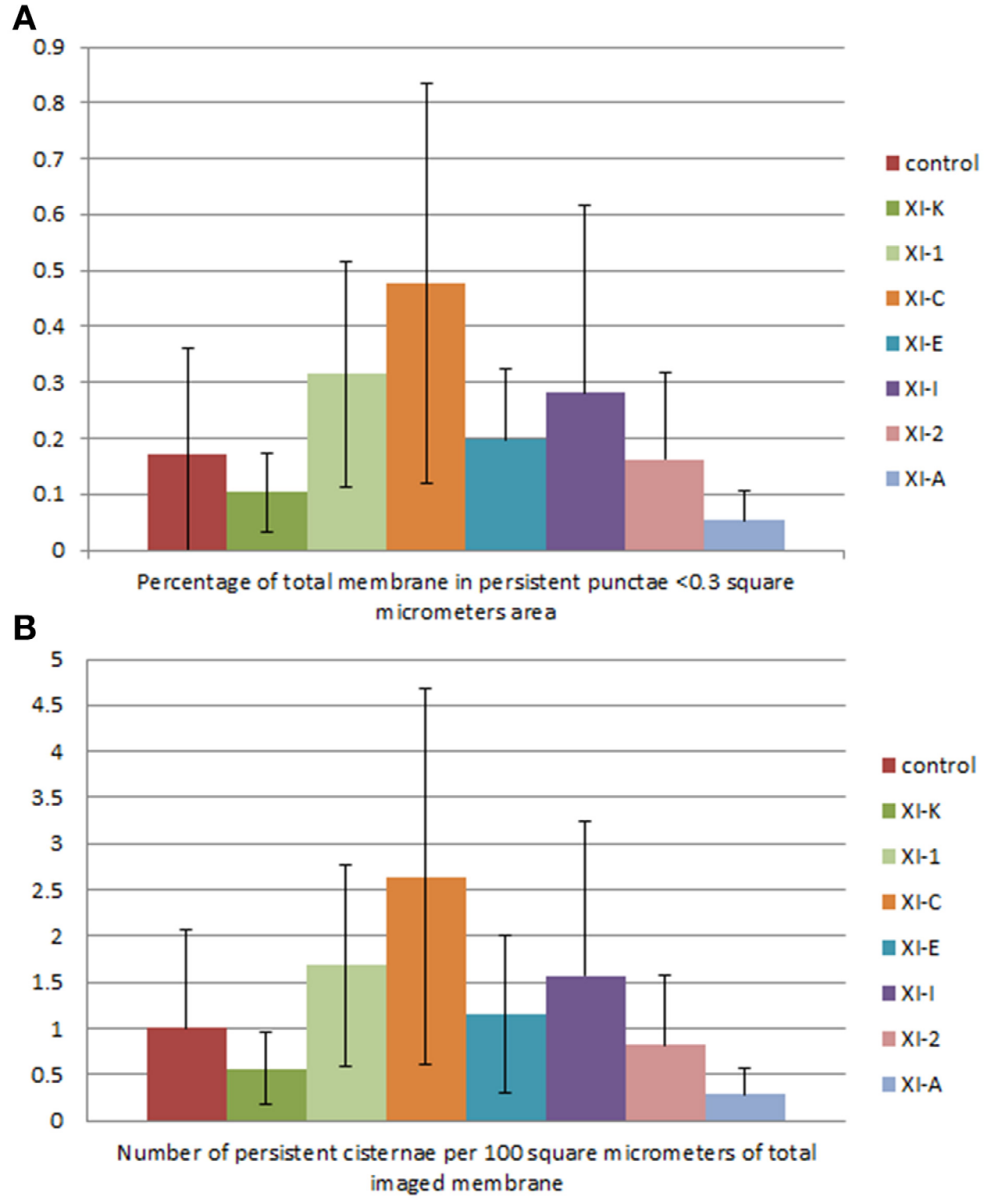
**Quantification of the effects of myosin tail domains on persistent ER punctae. (A)** Counts of the cumulative percentage area of highly persistent punctae (see Figures 5, 6 for examples). **(B)** Counts of the number of persistent punctae per hundred square micrometers of ER membrane analyzed for all of the myosin subclasses. Error bars represent standard deviation. The level of significance of the difference between the samples is described in Table 1.

In general, persistency mapping shows an increase in persistent ER tubules (Figures 2–4), cisternae (Figures 5–7), and puncta (Figures 5, 6, 8) in cells expressing certain myosin tail fusions compared to control cells. In all of the persistency map figures (Figures 2, 3, 5, 6) the image in the first column is the first frame from a representative movie and the additional columns highlight the process of isolating the highly persistent regions in the movie shown. These highly persistent regions (i.e., present for more than 23 s) are then quantified in terms of average number, size, and the percentage of imaged membrane represented by these morphological structures. Statistical analysis (HSD Tukeys test) of pair wise combinations of average values are shown in Table 1 (*n* = 15). Movies S1–S8 highlight the persistent ER elements in Figures 2, 3, 5, 6.

**Table 1:**
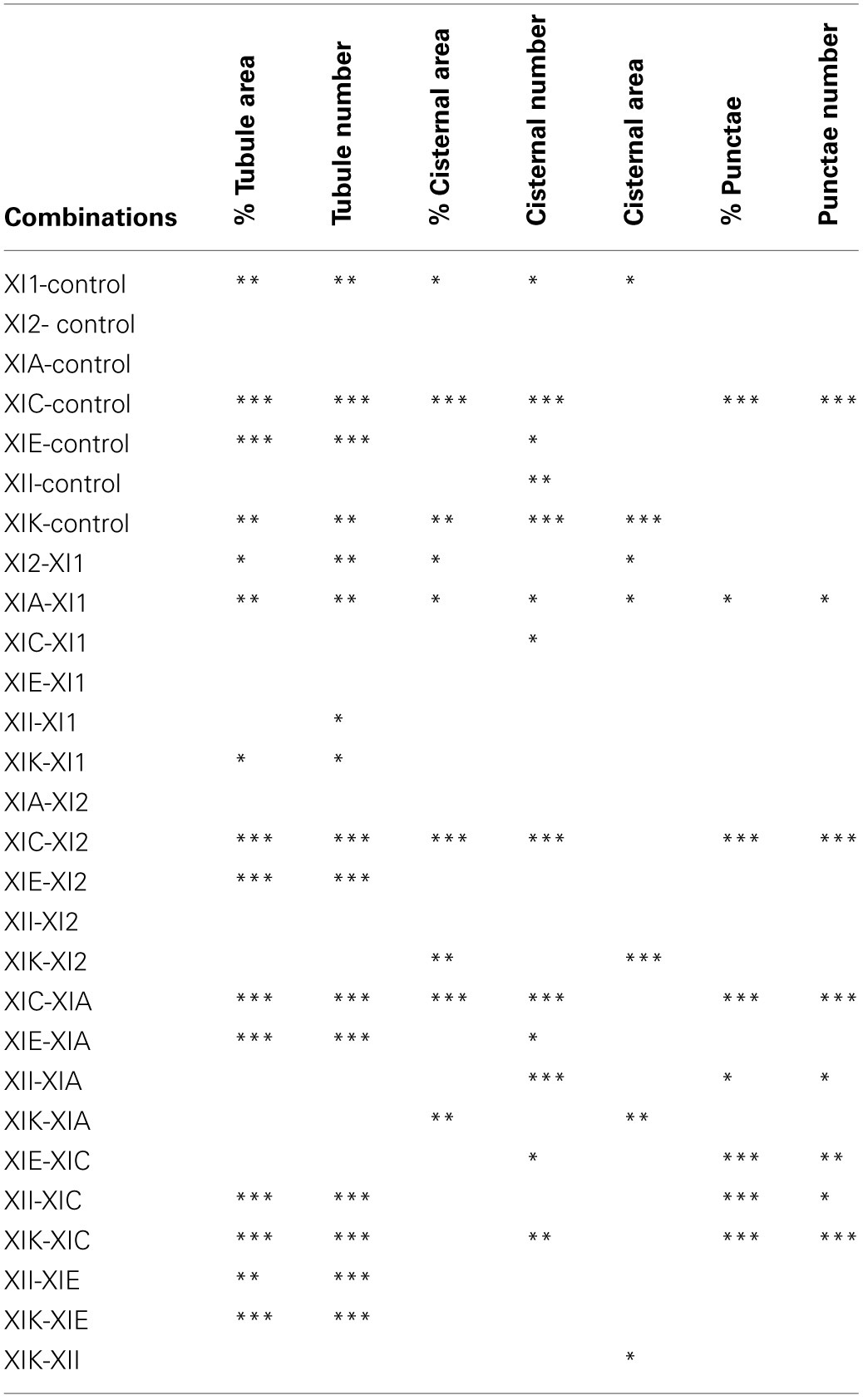
**Statistical analysis of persistency mapping**.

Tukeys HSD comparison of means test to compare persistent regions in the ER generated upon expression of myosin tail domains in tobacco leaf epidermal cells. Significant differences between pair wise combinations under test are shown (^*^p < 0.05; ^**^p < 0.005; ^***^p < 0.0005).

Whilst there is a large variation within the sample population (*n* = 15), statistical analysis highlighted that average persistent tubule area and number were significantly higher in cells coexpressing the tails of XI-C, XI-E, XI-K, or XI-1 compared to the control (Figures 2–4, Table 1). This corresponded with an increase in mesh size of the tubular network (Figure S2). In addition, there appeared to be significant pair wise differences between several of these myosins except for XI-C, XI-E, and XI-1 which are presumably performing similar functions relating to ER tubule dynamics/formation.

There was also a significant increase in the percentage of total membrane imaged present in persistent cisternal regions for several myosin tails (XI-1, XI-C, and XI-K) compared with the control (Figures 5–7, Table 1, a histogram of persistent cisternal size classes for each combination is provided Figure S3). These significant differences could relate to changes in either average size and/or number of persistent cisternae per given unit area. Both XI-1 and XI-K's effects were due to a combination of both increased number and average size of cisternal regions (Figure 7). XI-E and XI-I also resulted in significantly more cisternae. In addition the average area and percent area increased with XI-E and XI-I tail domain expression over control, but the data are so variable (note large standard deviations) that a firm statement of difference cannot be made. This high variability quite likely arises from the observations based on the movies, as described above, that cisternalization increases but a fairly large proportion remain motile. While the number of relatively immobile cisternae increases, there is enough general membrane cisternalization (hence larger amounts of membrane per field) that the area fraction of immobile cisternae does not reliably increase.

Pair wise statistical tests for significant differences between XI-1, XI-C, and XI-K persistent cisternal criteria indicate that XI-1 and XI-K are not different to one another. However, XI-C compared with either XI-1 or XI-K display differences in persistent cisternal number, but not persistent cisternal area. This could indicate that XI-1 and XI-K have conserved functions in generating the number and size of persistent ER cisternae, whereas XIC is more involved in maintaining cisternal number, but not size relative to the roles of XIK and XI-1.

Persistent regions of ER less than 0.3 μm^2^, termed persistent punctae, are present in control cells and in all cells coexpressing the myosin tail domains. XIC tail domain is the only myosin to have a significant increase in the number of persistent punctae over control conditions, and when compared with other myosins (Figures 5, 6, 8, Table 1).

Finally we tried to monitor the active regions of the ER network using displaced frame differences (Figure S4). Here, the sum of the displaced frame differences summed over the imaged volume were plotted for all myosin combinations. As expected with increased persistency for XI-K, XI-C, XI-1, and XI-E we have a slower rate of movement in these samples compared to the control, XI-2 and XI-A. This provides an initial approximation to the dynamic elements of the network.

## Discussion

ER network structure (tubules/cisternae and three way junctions) is not completely dependent on cytoskeletal elements; depolymerization of actin results in an interconnected ER network which is more cisternal (Sparkes et al., 2009a, 2011). At present, additional molecular factors required to alter ER morphology in plants include reticulons and RHD3 (see references in reviews Sparkes et al., 2011, and Zhang and Hu, 2013). Here, we have begun to assess the relative role(s) of class XI myosins on ER geometry and remodeling. Several class XI myosins which appear to have a global effect on perturbing the movement of several organelle classes including peroxisomes, Golgi and mitochondria were investigated (XI-C, XI-E, XI-I, XI-K, XI-1, and XI-2; (Avisar et al., 2008, 2009; Peremyslov et al., 2008, 2010; Prokhnevsky et al., 2008; Sparkes et al., 2008, 2009a). XIA was also tested as the tail domain fusion did not affect global organelle dynamics in previous studies, and so was used here as a potential control for overexpressing a class XI myosin.

The main findings from this study indicate that whilst XI-A did not affect ER dynamics, XI-C, XI-E, XI-K, and XI-1 affected tubule persistency and the persistent nature of cisternae, and XI-C increased the number of persistent punctae. Whilst XI-I and XI-2 didn't affect the persistent regions within the network, visual inspection indicates that XI-I increases the level of cisternalization, while XI-2 reduces overall remodeling (Movies S3, S4). These differential affects could indicate specific roles for myosins in controlling tubulation (growth/shrinkage/lateral sliding to form polygons), cisternalization, and potential anchoring of the ER to the plasma membrane. For example, based on the differences these myosin tail domains exert on static regions within the ER network, it is interesting to speculate that XI-C, XI-E, XI-K, and XI-1 are required for most forms of tubule growth and shrinkage as overexpression of the tail domains increased the number of static tubules. This is assuming that the tail domains are acting in a dominant negative manner and the observed effect is due to a direct rather than indirect effect. It is likely that tubule growth draws upon, in part, a reservoir of membrane in the cisternal regions of the ER rather than being due to extension of completely newly synthesized membrane (Sparkes et al., 2009a). Mesh size of the tubular network increases in those treatments that increase tubule persistency (Figures 2, 3 compared with Figure S2), indicating a lower amount of total tubulation. This explains why the myosin tail domains which increase the number of persistent tubules also have a concomitant increase in persistent cisternal size or number. The less obvious, but none-the-less significant effect, (Figure 7, Table 1) of XI-I increasing the number of persistent cisternae relative to control may relate to a shaping role in the ER of known cisternal structures, such as the nuclear envelope, where XI-I tends to accumulate (Figure 1). Hence, XI-I may have more shaping-related and less movement-related functions, while XI-C, XI-E, XI-K, and XI-1 may have more movement-related and less shaping-related functions. XI-2 tail domain expression doesn't affect the level of persistency in the ER, but appears to reduce overall modeling.

It is important that we comment upon the large variability seen within each sample population presented herein. Organelle dynamics are affected by growth and developmental cues, with larger, older cells showing faster ER and other organelle movement than smaller, younger cells (Stefano et al., 2014). The cell population analyzed here, the pavement epidermal cells between the minor veins of fully-expanded tobacco leaves, was chosen because it is amenable to rapid transient expression assays and provides easily-imaged, robust cells. However, this population varies somewhat in cell size and developmental stage. This could be one source of the variability. Other approaches, such as using myosin mutants, may provide analysis of a single developmental stage of a uniformly-sized cell population, but would suffer from the developmental defects caused by this mutation. Another source of variability in this work could arise from subcellular variations of movement within these puzzle-piece-shaped cells, which may have tip growth dynamics in the interdigitating lobes and non-tip growth dynamics along the plane of the outer wall. The pattern of cytoplasmic movement in cells undergoing polar tip vs. radial growth are radically different (Hepler et al., 2001). Unfortunately, technical limitations prevent acquiring data sets of adequate resolution to resolve the ER network in the entire cell within a fraction of a second. However, were that possible, different patterns of movement might be sorted and the ER dynamics within each pattern analyzed. Advances in imaging will hopefully overcome these limitations.

Previous studies using RNAi, expression of myosin truncations and observations in null mutant backgrounds have shown several myosins appear to have a global affect on the motility of all organelles tested (Avisar et al., 2008, 2009, 2012; Peremyslov et al., 2008, 2010; Prokhnevsky et al., 2008; Sparkes et al., 2008, 2009a). Interestingly, as detailed above, the same myosin truncations that globally arrest spheroid organelle movement have differential effects on ER dynamics and persistent regions. The effects of XI-I on spheroid dynamics could have been due to increased ER cisternalization reducing the effective void volume (cytoplasm) in which spheroid organelles could move thus reducing their motility. XI-2 reduces the movement of all organelles tested, including the ER to a certain extent, but doesn't affect the level of persistency. This could indicate that XI-2 affects a more global common element in organelle dynamics such as the actin cytoskeleton or perhaps cyclosis. Further examination of the nature of the change in the dynamics under control conditions, and in comparison with XI-2 tail domain expression (see Movie S3), awaits other forms of analysis. None-the-less, these results indicate that the myosin tail domains that globally affect the movement of spheroid organelles have differential effects on ER network dynamics; increased persistency (XI-1, XI-C, XI-K, XI-E) vs. reduced remodeling (XI-2) vs. drastic changes in ER cisternalization (XI-I).

The expression profiles of the myosins under study here indicate that XI-1, XI-2, XI-I, and XI-K are ubiquitously expressed throughout the plant, whereas XI-C and XI-E are mainly expressed in the stamen and pollen (Peremyslov et al., 2011; Sparkes et al., 2011). Unlike leaf epidermal cell, pollen tubes undergo polarized tip growth where organelle movement occurs via reverse fountain streaming (Hepler et al., 2001; Lovy-Wheeler et al., 2007). This type of growth and pattern of organelle movement could place additional demands on ER morphology; persistent punctae could possibly represent tethering/interaction with the plasma membrane to maintain geometric distribution throughout growth. Ridge et al. (1999) documented changes in ER morphology during developmental transitions in polarized root hair growth; upon completion of growth the ER is tubular whereas during the elongation phase a more cisternal form prevails some way behind the tubular ER network in the expansion zone at the tip.

Interestingly, based on phylogenetic analysis of the myosin motor domains it appears that XI-C, XI-E, XI-K, and XI-1 from *Arabidopsis* are from closely related subfamilies XI(J) and XI(K). This could therefore indicate that they might perform similar/conserved functions in controlling ER dynamics (Peremyslov et al., 2011). Whereas XI-A, XI-I, and XI-2 are from more distantly related subfamilies XI(G), and XI(I).

Myosins have been implicated in ER formation and/or dynamics in several plant systems. Immunoblotting of subcellular fractions from Arabidopsis leaf material indicated that XI-K cosedimented with an ER fraction, and that the streaming of the ER is drastically reduced in *xi-k* mutants and to a lesser extent in *xi-1* and *xi-2* (Ueda et al., 2010). Independent studies place XI-K on motile vesicles, and appears to interact and collocate with members from a novel myosin receptor family (Peremyslov et al., 2012, 2013). Moreover, immunolocalization studies indicated that XI-2 partially localized to peroxisomes in *Arabidopsis* (Hashimoto et al., 2005), whereas a 175 kDa tobacco homolog with 75% identity to XI-2 was proposed to control ER movement in tobacco BY-2 cells (Yokota et al., 2009). *In vitro* reconstitution experiments from tobacco BY-2 cells further supported a role for the 175 kDa myosin in ER tubule formation (Yokota et al., 2011). More recently, expression of the tail domain of a maize XI-I homolog Opaque1 in *N. benthamiana* disrupted ER streaming, and subcellular fractionation indicated it is also associated with the ER in maize (Wang et al., 2012). Interestingly, Ueda et al. (2010) also highlighted that actin organization is perturbed in *xi-k xi-2* double mutants, but not single mutants, indicating that both myosins act in a cooperative/synergistic manner to control actin organization in elongating cells. Defects in actin organization were also seen in the midvein cells of triple (*xi-k/1/2*) and quadruple (*xi-k/1/2/i*) Arabidopsis mutants (Peremyslov et al., 2010). However, actin dynamics rather than organization were affected in root hairs of an *xi-k* null line (Park and Nebenführ, 2013). In light of all of these studies, it is interesting to note that we found XI-2 may have a global affect on organelle dynamics which could be an indirect effect through perturbing actin organization. Therefore, organelle-myosin specificity, and the specific affect of myosins on actin dynamics, is still a matter of debate, and whether these differences relate to species or tissue specific effects is unclear. Determining the mechanism of myosin association and disassociation from targets is complex (Li and Nebenführ, 2007, 2008a,b). A recent publication from Peremyslov et al. (2013) highlighted a potential myosin receptor family in plants which will hopefully help resolve organelle-myosin specificity.

Persistency mapping is an excellent tool for quantifying the more static geometric elements within the dynamic ER network, which is continually undergoing rapid geometric rearrangements. It does not, however, monitor ER luminal or surface flow. Prior work (Sparkes et al., 2009a) has shown that non-directional surface flow as measured with FRAP and photoactivation still occurs when actin is depolymerized, a condition that drastically changes ER geometry. Optical flow analysis of fluorescent ER constituent proteins also shows that actin depolymerization does not completely inhibit movement (Stefano et al., 2014). In single mutant *xi-1*, there is only a small change in ER movement as analyzed with optical flow (Ueda et al., 2010), but XI-1 tail domain expression has a large effect on membrane geometry (Figures 1, 2, 4, 5, 7). Persistency mapping therefore provides the only tool currently available to quantify differences in ER network geometry. Further studies are required to determine whether the same myosins which affect ER network dynamics also affect surface flow. Better analytical tools are required to quantify the complex dynamic regions which consist of tubule growth, shrinkage, lateral sliding, changes from tubular to cisternal form, formation and movement of three way junctions, polygon formation, size, movement and potential filling to form cisternae. Whilst these dynamic elements can be broken down into various aforementioned categories, ER remodeling is a complex combination where each morphological element is interdependent requiring a more sophisticated cross correlative approach. Our initial displaced frame difference provides an initial observation of such dynamic elements. Furthermore, high persistency described here does not distinguish between elements which are static over sequential frames lasting more than 23 s vs. temporally episodic events over a similar discontinuous time frame. However, observation of the movies clearly shows that persistent elements are consistently static rather than being due to events which reoccur. In addition, it is worthwhile noting that whilst the studies presented here relate to real time events, they are limited by the spatial resolution of the microscope. In essence, the ER tubules studied are likely to be around the 50–70 nm diameter making the 200 nm resolution limit of the microscope prone to potentially imaging parallel tubules rather than reflecting single tubules. Only through the development of super resolution imaging systems, which allow imaging in real time, will these types of issues be resolved.

## Author contributions

Imogen Sparkes designed the experiments, Lawrence R. Griffing carried out the persistency mapping and statistical analysis, Imogen Sparkes, Lawrence R. Griffing, and Hongbo T. Gao acquired the data. All authors were involved in writing and approving the manuscript.

### Conflict of interest statement

The authors declare that the research was conducted in the absence of any commercial or financial relationships that could be construed as a potential conflict of interest.

## Supplementary material

Figure S1 **Western blot analysis of transiently expressed mRFP-myosin tail fusions in tobacco leaf epidermal cells**. Arrow heads denote expected sizes of full length mRFP myosin tail fusions (~100 kDa apart from XI-A which is ~124 kDa). Asterisk shows detection of full length St-mRFP used as a control. Differences in relative myosin levels are due to differences in the level of transient expression. A large proportion of each myosin fusion is stable.

Figure S2 **Mesh number per estimated cytoplasmic volume and ER mesh size**. The ER tubules generate open polygons that can be considered as elements of a mesh. Blue bars: The polygonal regions subtended by tubules were counted for each frame in each movie, averaged over the entire movie, divided by the estimated cytoplasmic volume, and shown as the average number of meshes per 300 μm^3^ of cytoplasm for all of the movies taken for each treatment. XI-1, XI-C, XI-E, XI-I, and XI-K are all significantly different to the control and XI-A (*p* < 0.005; Tukeys HSD comparison of means). Red bars: The average area, in square micrometers, of each polygonal mesh structure for each frame of each movie was also calculated. The average mesh area over all of the movies of each treatment is shown. XI-C, XI-E, and XI-K are all significantly different to the control (*p* < 0.005; Tukeys HSD comparison of means). The mesh areas of XI-K and XI-E were also significantly different (*p* < 0.05) from XI-2, XI-A, and XI-I. Error bars are standard deviation.

Figure S3 **Histogram of the percent of each persistent cisternae in different size classes for each treatment**. The distribution of the percentage that each different size class makes to the population of persistent cisternae produced by each treatment is shown. The value of each size class was chosen so that comparisons could be made between those that had predominantly small cisternae (e.g., control) and those that had very large cisternae (e.g., XI-1 and XI-K).

Figure S4 **Relative movement or flow as shown by average displaced frame differences summed over imaged volumes**. The value of the intensity difference between every fifth frame, the displaced frame difference, is summed for each movie and divided by the approximate volume of the region imaged (area of signal in a 3 μm optical section). The average of these values is shown for all of the movies acquired for each treatment. Error bars are standard deviation. XI-1, XI-C, XI-E, XI-K, and XI-I are significantly different to the control (*p* < 0.005; Tukeys HSD comparison of means), and to XI-A (*p* < 0.05; Tukeys HSD comparison of means), and XI-1, XI-C, XI-E, and XI-K are significantly different to XI-2 (*p* < 0.05; Tukeys HSD comparison of means).

Movie S1 **Persistency mapping of ER dynamics in control tobacco leaf epidermal cells**. Time lapse movie showing the movement of the ER, along with overlays of the persistent tubules, cisternae, and puncta (magenta). Scale bar 10 μm.

Movie S2 **Persistency mapping of ER dynamics in a tobacco leaf epidermal cell expressing the myosin tail domain XI-A**. Time lapse movie of the ER in a cell coexpressing the tail domain of XI-A. Overlays of the persistent tubules, cisternae and puncta (magenta) are also shown for comparison. Scale bar 10 μm.

Movie S3 **Persistency mapping of ER dynamics in a tobacco leaf epidermal cell expressing the myosin tail domain XI-2**. Time lapse movie of the ER in a cell coexpressing the tail domain of XI-2. Overlays of the persistent tubules, cisternae and puncta (magenta) are also shown for comparison. Scale bar 10 μm.

Movie S4 **Persistency mapping of ER dynamics in a tobacco leaf epidermal cell expressing the myosin tail domain XI-I**. Time lapse movie of the ER in a cell coexpressing the tail domain of XI-I. Overlays of the persistent tubules, cisternae and puncta (magenta) are also shown for comparison. Scale bar 10 μm.

Movie S5 **Persistency mapping of ER dynamics in a tobacco leaf epidermal cell expressing the myosin tail domain XI-C**. Time lapse movie of the ER in a cell coexpressing the tail domain of XI-C. Overlays of the persistent tubules, cisternae and puncta (magenta) are also shown for comparison. Scale bar 10 μm.

Movie S6 **Persistency mapping of ER dynamics in a tobacco leaf epidermal cell expressing the myosin tail domain XI-E**. Time lapse movie of the ER in a cell coexpressing the tail domain of XI-E. Overlays of the persistent tubules, cisternae and puncta (magenta) are also shown for comparison. Scale bar 10 μm.

Movie S7 **Persistency mapping of ER dynamics in a tobacco leaf epidermal cell expressing the myosin tail domain XI-K**. Time lapse movie of the ER in a cell coexpressing the tail domain of XI-K. Overlays of the persistent tubules, cisternae and puncta (magenta) are also shown for comparison. Scale bar 10 μm.

Movie S8 **Persistency mapping of ER dynamics in a tobacco leaf epidermal cell expressing the myosin tail domain XI-1**. Time lapse movie of the ER in a cell coexpressing the tail domain of XI-1. Overlays of the persistent tubules, cisternae and puncta (magenta) are also shown for comparison. Scale bar 10 μm.

Movie S9 **Comparison of ER dynamics in two tobacco leaf epidermal cells expressing relatively different levels of mRFP-XI-1 tail domain fusion**. Fluorescent image highlighting mRFP-XI-1 tail fusion (magenta) and the effects on ER dynamics in the same cell are shown (green). Two representative movies from two cells show similar dynamics in cells with relatively different levels of myosin tail expression.
