# Corrigendum: ER network dynamics are differentially controlled by myosins XI-K, XI-C, XI-E, XI-I, XI-1, and XI-2

**DOI:** 10.3389/fpls.2014.00637

**Published:** 2014-11-12

**Authors:** Hong-Bo Gao

**Affiliations:** Biosciences, CLES, Exeter UniversityExeter, UK

**Keywords:** endoplasmicreticulum, movement, myosin, persistencymapping, dynamics, remodeling

In the article “ER network dynamics are differentially controlled by myosins XI-K, XI-C, XI-E, XI-I, XI-1, and XI-2,” published 21 May 2014, with myself as the second author has a mis-spelling/ mis-formatting of my name.

Currently my name is spelled as: Hongbo T. Gao.

The correct spelling is: Hong-Bo Gao (Initial, H. B. Gao). I did not notice this mistake during the review process.

## Conflict of interest statement

The author declares that the research was conducted in the absence of any commercial or financial relationships that could be construed as a potential conflict of interest.

