# Supplementary figures and images for: ER network dynamics are differentially controlled by myosins XI-K, XI-C, XI-E, XI-I, XI-1, and XI-2
Source: Front Plant Sci. 2014 May 21;5:218. doi: 10.3389/fpls.2014.00218 (PMC4033215; doi:10.3389/fpls.2014.00218)

ST-RFP XI-K XI-1 XI-C XI-E XI-I XI-2 XI-A

175

80

58

46

30

23

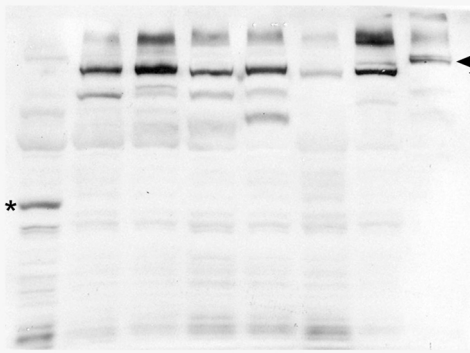

Griffing et al. Supp. Fig 1

Supplement: Figure S1 — Western blot analysis of transiently expressed mRFP-myosin tail fusions in tobacco leaf epidermal cells. Arrow heads denote expected sizes of full length mRFP myosin tail fusions (~100 kDa apart from XI-A which is ~124 kDa). Asterisk shows detection of full length St-mRFP used as a control. Differences in relative myosin levels are due to differences in the level of transient expression. A large proportion of each myosin fusion is stable. [file Presentation1.PDF]

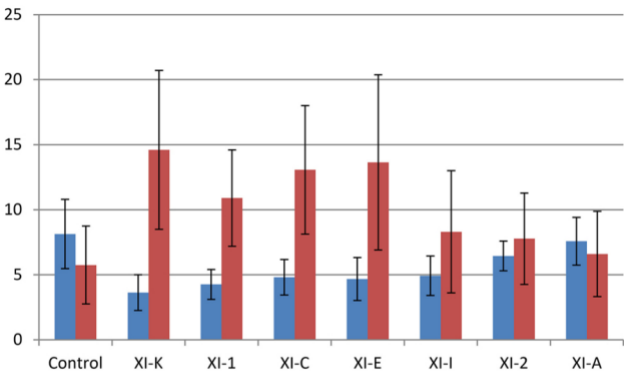

Griffing et al Supp. fig 2

Supplement: Figure S2 — Mesh number per estimated cytoplasmic volume and ER mesh size. The ER tubules generate open polygons that can be considered as elements of a mesh. Blue bars: The polygonal regions subtended by tubules were counted for each frame in each movie, averaged over the entire movie, divided by the estimated cytoplasmic volume, and shown as the average number of meshes per 300 μm3 of cytoplasm for all of the movies taken for each treatment. XI-1, XI-C, XI-E, XI-I, and XI-K are all significantly different to the control and XI-A (p < 0.005; Tukeys HSD comparison of means). Red bars: The average area, in square micrometers, of each polygonal mesh structure for each frame of each movie was also calculated. The average mesh area over all of the movies of each treatment is shown. XI-C, XI-E, and XI-K are all significantly different to the control (p < 0.005; Tukeys HSD comparison of means). The mesh areas of XI-K and XI-E were also significantly different (p < 0.05) from XI-2, XI-A, and XI-I. Error bars are standard deviation. [file Presentation2.PDF]

Percentage of persistent  
cisternae in each size class

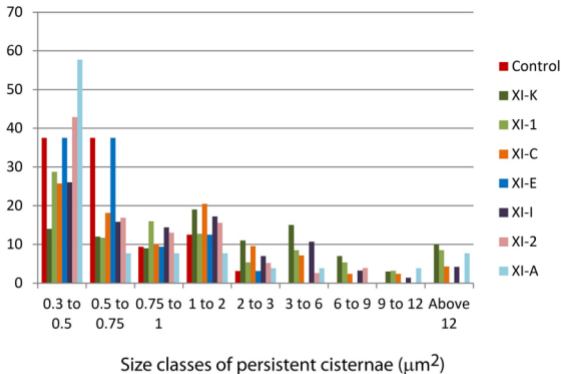

Supplement: Figure S3 — Histogram of the percent of each persistent cisternae in different size classes for each treatment. The distribution of the percentage that each different size class makes to the population of persistent cisternae produced by each treatment is shown. The value of each size class was chosen so that comparisons could be made between those that had predominantly small cisternae (e.g., control) and those that had very large cisternae (e.g., XI-1 and XI-K). [file Presentation3.PDF]

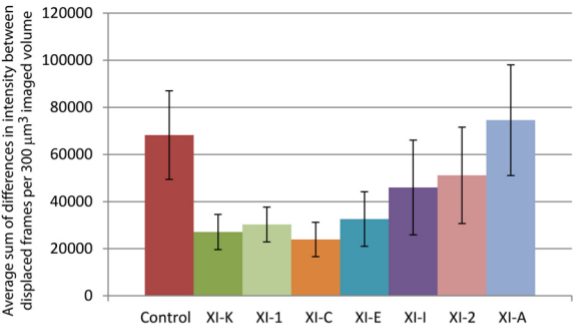

Griffing et al. Supplemental Figure 4

Supplement: Figure S4 — Relative movement or flow as shown by average displaced frame differences summed over imaged volumes. The value of the intensity difference between every fifth frame, the displaced frame difference, is summed for each movie and divided by the approximate volume of the region imaged (area of signal in a 3 μm optical section). The average of these values is shown for all of the movies acquired for each treatment. Error bars are standard deviation. XI-1, XI-C, XI-E, XI-K, and XI-I are significantly different to the control (p < 0.005; Tukeys HSD comparison of means), and to XI-A (p < 0.05; Tukeys HSD comparison of means), and XI-1, XI-C, XI-E, and XI-K are significantly different to XI-2 (p < 0.05; Tukeys HSD comparison of means). [file Presentation4.PDF]
